# Vibration Emissions Reduce Boar Sperm Quality via Disrupting Its Metabolism

**DOI:** 10.3390/biology13060370

**Published:** 2024-05-23

**Authors:** Shanpeng Wang, Xuejun Zeng, Shenao Liu, S. A. Masudul Hoque, Lingjiang Min, Nengshui Ding, Zhendong Zhu

**Affiliations:** 1 College of Animal Science and Technology, Qingdao Agricultural University, Qingdao 266109, Chinamljlab405@qau.edu.cn (L.M.); 2 Fujian Aoxin Biotechnology Group Co., Ltd., Zhangzhou 363000, China; sdmy2009@yeah.net; 3 Ji’an Aobao Biotechnology Group Co., Ltd., Ji’an 343000, China; 4 Department of Animal Breeding and Genetics, Bangabandhu Sheikh Mujibur Rahman Agricultural University, Gazipur 1706, Bangladesh; mhoqueabg@bsmrau.edu.bd; 5 State Key Laboratory for Pig Genetic Improvement and Production Technology, Jiangxi Agricultural University, Nanchang 330045, China

**Keywords:** boar sperm, metabolic, vibrations, sperm function, transport

## Abstract

**Simple Summary:**

Boar sperm is a highly sensitive biological product, and its quality is susceptible to various environmental factors during transportation. Presently, vibration emissions were observed and documented during the transportation of artificial insemination doses. Vibrations, serving as an environmental stressor, harbor the potential to exert an influence on sperm quality. However, the precise mechanism underlying the detrimental effects of vibration emissions on sperm functionality remains elusive. In the present research, we discerned that vibration emissions impair the quality of boar sperm by modulating their metabolic processes, ultimately resulting in a diminished fertilization capability.

**Abstract:**

Artificial insemination (AI) with liquid-preserved semen has recently become common in pig breeding. The semen doses are produced in a centralized manner at the boar stud and then subsequently distributed and transported to sow farms. However, vibration emissions during transportation by logistic vehicles may adversely affect the quality of boar sperm. Therefore, this study aimed to explore the impact of vibration-induced emissions on sperm quality and function under simulated transportation conditions. Each time, ejaculates from all 15 boars were collected and then pooled together to minimize individual variations, and the sample was split using an extender for dilution. Different rotational speeds (0 rpm, 80 rpm, 140 rpm, 200 rpm) were utilized to simulate varying intensities of vibration exposure using an orbital shaker, considering different transportation times (0 h, 3 h, and 6 h). Subsequently, evaluations were conducted regarding sperm motility, plasma membrane integrity, acrosome integrity, mitochondrial function, adenosine triphosphate (ATP) levels, mitochondrial reactive oxygen species (ROS) levels, pH, glycolytic pathway enzyme activities, and capacitation following exposure to vibration emissions. Both vibration time and intensity impact sperm motility, plasma membrane integrity, and acrosomal integrity. Vibration exposure significantly reduced sperm ATP levels, mitochondrial membrane potential, and the levels of mitochondria-encoded proteins (MT-ND1, MT-ND6) (*p* < 0.05). After vibration emission treatment, the pH value and mitochondrial ROS levels significantly increased (*p* < 0.05). Inhibition of sperm glycolysis was observed, with reduced activities of hexokinase (HK), pyruvate kinase (PK), and lactate dehydrogenase (LDH), along with decreased lactate levels (*p* < 0.05). Additionally, sperm tyrosine phosphorylation levels were significantly reduced by vibration emissions compared to the control group (*p* < 0.05). After the vibration emission treatment, the number of sperm bound to each square millimeter of oviduct explants decreased significantly compared to the control group (*p* < 0.05). Similarly, compared to the control group, using semen subjected to vibration stress for AI results in significantly reduced pregnancy rates, total born litter size, live-born litter size, and healthy born litter size (*p* < 0.05).

## 1. Introduction

Effective management of a breeding herd is contingent upon successful breeding practices. Artificial insemination (AI) is a pivotal tool in pig reproduction, meeting the rising demand for high-quality pork and enhancing herd health [1]. As AI technology has progressed, semen production units have been centralized, necessitating the transportation of semen doses over extended distances via logistics vehicles within contemporary pig-breeding systems [2,3]. This transportation process from semen production units to sow farms after collection and processing typically spans hours to days [4]. However, past studies have revealed that boar sperm was notably susceptible to the stresses linked to handling and transport, resulting in sublethal cellular harm [5] and diminished viability [6]. Furthermore, several studies indicated that temperature fluctuations [7], UV light [3], and vibrations [4,8,9,10] could adversely affect boar sperm quality. Nonetheless, the specific impact of vibration emissions on boar sperm function during the transportation process remains unclear.

Vibration, as an environmental stressor, possesses substantial potential to influence the reproductive system [11,12,13]. Previous studies have demonstrated that long-term exposure to whole-body vibration diminished sperm quality, with reduced sperm motility and an increased rate of sperm deformation [14]. During driving, vibrations are typically correlated with activities like acceleration, steering, and braking [15]. Hafemeister et al. (2022) [3] reported that both speed and road surfaces significantly influenced vibration emissions in standardized road trials. In addition, in various industries, including the transport of fruit and beverages, it is imperative to minimize vibration emissions to maintain product quality [16,17,18]. In boar sperm, when the semen dose was under shaking stress, the percentage of sperm viability, acrosome integrity, and plasma membrane integrity exhibited a negative correlation with vibration emissions [8]. Moreover, Tamanini et al. (2022) observed reduced sperm mitochondrial activity when boar sperm was exposed to an orbital shaker at 70 rpm [9]. Thus, mitigation of the damage caused by vibration emissions becomes essential to preserving sperm quality.

Research suggests that shear stress induced by vibration emissions and the interaction between cells and the gas–liquid interface may be factors contributing to the reduction in cellular vitality [19]. It is well known that the agitation of semen generates substantial foam during dilution or transport [4]. During the production of semen doses, AI centers usually try to minimize the formation of bubbles, since the forces produced during these events as well as the number and size of bubbles are positively correlated with cell damage [19]. Moreover, the generation of bubbles and foam leads to alterations in the liquid’s pH [20]. Previous reports have indicated that boar sperm motility decreases as pH levels rise [21], and adverse effects are observed when the pH exceeds 7.5 [22]. Therefore, alkalinization of the extender solution, induced by vibration emissions, is proposed to cause reduced sperm motility [8]. The imbalance between reactive oxygen species (ROS) generation and antioxidant defense mechanisms may give rise to cellular damage and oxidative stress [23]. Excessive ROS production could also potentially be one of the contributing factors to the diminished sperm vitality observed following vibration emission treatment. Multiple studies have demonstrated that excessive ROS production can impair sperm motility [24,25] and damage the mitochondrial gene expression system, leading to reduced ATP production [26]. Paschoal et al. (2021) found that exposure to vibration emissions significantly increased ROS levels in sperm, resulting in sperm experiencing oxidative stress [4]. However, research on the impact of vibration emissions on sperm remains limited thus far.

Sperm motility is contingent upon a highly efficient energy metabolism. In order to uphold homeostasis and motility, sperm cells exhibit the capacity to dynamically switch between distinct metabolic pathways [27]. Prior investigations have unequivocally highlighted the pivotal role of glycolysis and mitochondrial functions in sustaining the functional integrity of mammalian sperm [28,29,30,31,32]. Although recent research has focused on the impact of vibration emissions on boar sperm, the precise mechanisms by which these emissions impair sperm function remain uncertain. Therefore, it is hypothesized that the stress induced by vibration during the transportation of AI doses may disrupt boar sperm metabolism and, subsequently, affect its functionality.

## 2. Materials and Methods

### 2.1. Experimental Design

Experiment 1 aimed to collect vibration data during semen transportation, simulate different levels of vibration using an orbital shaker, and investigate the effects of different speeds and treatment times on the quality of boar sperm. In this study, 15 mature and fertile Large White boars aged 17.4 ± 0.74 (±SD) months were used to collect semen. Each time, ejaculates were collected from all 15 boars and then pooled together to minimize individual variations. Only ejaculates with more than 90% total sperm motility and a minimum of 85% morphology normal sperm were used in this study. The mixed raw semen was diluted with an extender (ZENOLONG, Taizhou, China) at a concentration of 3.0 × 10^7^ sperm/mL and then divided into 12 parts (at 4 different speeds (0 rpm, 80 rpm, 140 rpm, and 200 rpm) and 3 points of time (0 h, 3 h, and 6 h)) and filled in 90 mL tubes. The filling volume was 80 mL of extended semen. Dilution was performed in one step, isothermally at 32 °C. The temperature of the extended semen was gradually lowered to prevent thermal shock. Following processing, the extended semen doses were transported to the laboratory in a temperature-controlled container set at 17 °C. To simulate the vibration emissions during the transportation of boar semen doses, three orbital shakers (WD-9405B, LiuYi Biology, Shanghai, China) were utilized, rotating at speeds of 80 rpm, 140 rpm, and 200 rpm, running at circular horizontal frequencies. The rotation speed was determined according to the range of vibration emission fluctuations during the actual transport. Boar semen was placed horizontally on an orbital shaker in an incubator of 17 ± 1 °C to simulate vibration emissions up to 6 h. The control semen dose was kept at 17 °C without vibration emissions. The semen doses were analyzed for motility, plasma membrane integrity, and acrosome integrity. To avoid exposure to air, separate semen doses were used at each assessment point. The results of Experiment 1 revealed a significant decrease in total motility, progressive motility, acrosome integrity, and plasma membrane integrity in the treatment group with a speed of 200 rpm. Therefore, this treatment group was selected for subsequent experiments.

Experiment 2 aims to investigate the effects of vibration emissions on boar sperm during semen transportation, simulated by a speed of 200 rpm to replicate vibration emissions. This experiment will explore the impact of vibration on boar sperm morphology, semen pH, mitochondrial function, glycolytic pathways, sperm capacitation, sperm binding to oviduct explants, and reproductive performance. Additionally, due to the greater impairment observed in sperm progressive motility and acrosome integrity after 6 h of treatment at 200 rpm and the sperm binding index (BI), vibration emission treatment was much lower in the in 6 h group than that in 3 h vibration emission treatment or the control group. Only semen treated for 3 h was utilized to assess its effects on reproductive performance in Experiment 2.

### 2.2. Chemicals

All chemicals and reagents were purchased from Sigma (Shanghai, China) unless specified otherwise.

### 2.3. Collection and Analysis of Vibration Data

To measure the actual semen transportation process and the vibrational data simulated by the orbital shaker, we employed a vibration sensor (DT-178A, CEM, Shenzhen, China) to record the vibrational data. This sensor recorded events related to semen transportation and simulated activities of the orbital shaker by measuring three-axis acceleration (x, y, z).

In the processing of raw data, we calculate the distance D as the vector length between two measurement points, p_m_ and p_n_, denoted as (Equation (1)):

(1) $$D=\left|\bar{p_{n}p_{m}}\right|=\sqrt{{\left(x_{n}-x_{m}\right)}^{2}+{\left(y_{n}-y_{m}\right)}^{2}+{\left(z_{n}-z_{m}\right)}^{2}}$$

These measurement points are described by three-axis acceleration data (x, y, z) at consecutive times m and n, representing the spatial displacement changes between two consecutive time points, used to indicate the vibration intensity.

To collect vibration data under real semen transport conditions, we simulated a 1500 km delivery of a dose of AI from a Chinese boar stud and recorded vibration events throughout the entire journey. The sampling rate for all recordings was set at a fixed value of 2 Hz (one sensor sample every 2 s). Vibration sensors were placed inside the semen transport container, alongside the transported AI doses. This transport was conducted using a transportation vehicle. Experimental conditions for collecting vibration sensor data with laboratory settings simulating vibration emissions during road transport were achieved using an orbital shaker at speeds of 0, 80, 140, and 200 rpm.

### 2.4. Animals and Semen Collection

This study was conducted from May 2022 to June 2023. Ejaculate was collected from 15 mature and fertile Large White boars aged 17.4 ± 0.74 (±SD) months. The ejaculate was collected by the gloved-hand technique. The pre-spermatic phase was discarded, and the gel fraction was removed by double-gauze filtration. The animals were housed in a commercial boar station located in Qingdao, Shandong Province, China. All boars were kept in individual pens and received commercial feed, with ad libitum access to water. All procedures involving the animals and experiments were conducted with the approval of the Qingdao Agricultural University Institutional Animal Care and Use Committee (QAU-1121010).

### 2.5. Evaluation of Sperm Motility Parameters

According to our previous study [33], sperm motility was promptly assessed after vibration emission treatment using a computer-assisted semen analysis (CASA) system (HT CASA-Ceros II; Hamilton Thome, MA, USA). The processed semen was then stored at 17 °C for up to 96 h, with sperm motility assessed daily. Briefly, 5 μL of semen sample was placed on a pre-warmed glass slide and randomly evaluated with three fields for motility detection.

### 2.6. Evaluation of Sperm Plasma Membrane Integrity and Acrosome Integrity

Following exposure to vibration-induced stress, the plasma membrane integrity and acrosome integrity of sperm were immediately analyzed using the LIVE/DEAD Sperm Viability Kit (Invitrogen™, Shanghai, China) for plasma membrane integrity and fluorescein isothiocyanate-peanut agglutinin (FITC-PNA, Sigma-Aldrich, Shanghai, China) for acrosomal integrity evaluation. To evaluate plasma membrane integrity, a mixture of 0.2 μL of 100 nM SYBR-14 and 0.5 μL of 2.4 mM PI working solution was added to 100 μL of the semen sample, followed by a 10 min incubation at 37 °C in the dark before analysis.

To assess acrosome integrity, 30 µL of the sperm sample was applied to a glass slide and fixed using methanol. The fixed sperm were subsequently incubated with 20 μL of a 100 µg/mL FITC-PNA solution for 30 min at 37 °C in the dark and further co-incubated with 20 μL of 2.4 mM PI for an additional 10 min before analysis. The plasma membrane integrity and acrosome integrity of the sperm were evaluated using a fluorescence microscope (ZEISS DM200LED, Oberkochen, Germany) with a 400× filter. Five random fields were selected, with each field containing more than 200 sperm, following the protocol of the previous study [34].

### 2.7. Measurement of Sperm ATP Level

Sperm ATP level was measured using an ATP Assay Kit (A095-1-1, Nanjing Jiancheng Bioengineering Institute, Nanjing, China) according to the manufacturer’s instructions. Briefly, sperm samples were lysed and centrifuged, and 20 μL of sperm supernatant was mixed with 180 μL of working solution in a 96-well plate. The absorbance was measured at 340 nm using a microplate reader (TECAN, Infinite M Nano, Männedorf, Switzerland).

### 2.8. Evaluation of Sperm Mitochondrial Membrane Potential

Changes in sperm mitochondrial activity were evaluated with the JC-1 Mitochondrial Membrane Potential Detection Kit (Beyotime Institute of Biotechnology, Shanghai, China), according to a previous study [35]. In this study, only the mitochondrial membrane potential of the sperm treated with a rotation speed of 200 rpm was assessed. Briefly, post-vibration emission treatment, sperm samples of 100 µL were immediately taken and stained with 100 µL 1× JC-1 working solution and incubated for 30 min at 37 °C in the dark. Samples were then centrifuged at 600× *g* for 3 min at 4 °C. Subsequently, samples were washed twice with JC-1 buffer and resuspended. The stained samples were evaluated by flow cytometer (FACSAria III, BD Biosciences, Franklin Lakes, NJ, USA). A total of 20,000 sperm events were analyzed.

### 2.9. Analysis of Hexokinase Activity and Pyruvate Kinase Activity

Sperm HK activity and PK activity were measured by HK assay kit (A077-3-1, Nanjing Jiancheng Bioengineering Institute, Nanjing, China) and PK assay kit (A076-1-1), respectively. According to the manufacturer’s instructions, sperm samples were crushed by ultrasound (ice bath, power 200 W, sonication 3 s, interval 10 s, repeated 30 times) and centrifuged at 8000× *g* for 10 min at 4 °C. The supernatant was collected and placed on ice for testing. The sperm supernatant was then mixed with a working solution in a 96-well plate to evaluate the activities of HK and PK with a microplate reader at 340 nm.

### 2.10. Measurement of Lactate Dehydrogenase Activity

LDH activity was measured using the LDH Assay Kit (A020-2-2), according to a previous study [36]. Briefly, sperm samples were crushed by ultrasound (ice bath, power 200 W, sonication 3 s, interval 10 s, repeated 30 times) and centrifuged at 8000× *g* for 10 min at 4 °C. The supernatant was collected and placed on ice for testing. The sperm supernatant was mixed with the working solution on a 96-well plate and left to stand for 5 min at room temperature, and LDH activity was measured at 340 nm using a microplate reader.

### 2.11. Detecting Lactic Acid Levels

A lactic acid assay kit (A019-2-1) was used to detect the lactic acid content in sperm, according to the manufacturer’s instructions [36]. Briefly, lactate levels were measured at 530 nm using a microplate reader by detecting the purple substance formed after NBT reduction by NADH.

### 2.12. Western Blotting

Sperm samples were lysed by sonication, and total sperm proteins were extracted with sodium dodecyl sulfate (SDS) sample buffer. Each sample was separated from the total proteins by 10% SDS-PAGE gel (EC0O23-B, Sparkjade, Jinan, China) and transferred to polyvinylidene fluoride (PVDF) membranes. Non-specific binding sites were blocked with 5% (*m*/*v*) bovine serum albumin diluted in tris-buffered saline with tween (TBST) (1% tris-buffered saline (TBS), 0.1% Tween 20). The membranes were immunoblotted with primary antibodies (MT-ND1, A17967, 1:1000, AB clonal, Wuhan, China; MT-ND6 Rabbit pAb, A17991, 1:1000, AB clonal; α-tubulin, AC008, 1:1000, AB clonal; Rabbit Anti-Phospho-Tyrosine, bs-10497R, 1:1000, Bioss, Beijing, China) diluted in 5% bovine serum albumin (BSA) overnight at 4 °C. After washing with TBST, the membrane was incubated with secondary antibodies (AS014, 1:1000, AB clonal, Wuhan, China) for 1 h. After washing with TBST three times, ECL plus (ED0016-B, Sparkjade, Jinan, China) was used to detect imprints through a gel imaging analyzer.

### 2.13. Sperm Morphology

To assess sperm morphology, 30 μL of sperm samples were spread on glass slides. After drying, the samples were fixed with formaldehyde. Subsequently, the slides were rinsed with water and stained with a 10% eosin solution (E607321-0100, BBI, Shanghai China). After staining, the slides were washed and dried, and 500 sperm samples were evaluated under a microscope (ZEISS DM20OLED, Oberkochen, Germany). Morphological assessment included normal sperm, abnormal head morphology, attachment of proximal and distal cytoplasmic droplets, abnormal neck morphology, and folding and coiling of the tail.

### 2.14. Monitoring of pH

After simulated vibration emission treatment, the pH of the boar semen was measured using a pH meter (PB-10, Saiduolisi, Beijing, China). The pH meter was previously calibrated with a standard solution (pH value = 6.86).

### 2.15. Measure of Sperm Mitochondrial ROS Level

A MitoSOX™ Red Assay Kit (M36008, Thermo Fisher Scientific, Shanghai, China) was used to detect the intracellular generation of sperm mitochondrial ROS. Briefly, sperm samples were incubated with 400 μL working solution (5 μM) at 37 °C for 15 min in the dark. The mitochondrial ROS level was evaluated by flow cytometer (FACSAria III, BD Biosciences, Franklin Lakes, NJ, USA) and measured as the mean fluorescence intensity (MFI). A total of 20,000 sperm events were analyzed.

### 2.16. Sperm Capacitation

After the simulated vibration emission treatment, sperm were exposed to a capacitation medium for 3 h at 38 °C. The capacitation medium consisted of 95 mM NaCl, 4.8 mM KCl, 1.2 mM KH_2_PO_4_, 5.55 mM glucose, 25 mM NaHCO_3_, 2 mM CaCl_2_, 2 mM sodium pyruvate, and 0.4% BSA, with the pH adjusted to 7.4 [37].

### 2.17. Sperm Binding in the Oviduct Explant

The in vitro binding capacity of sperm to oviduct epithelial cells was assessed as described in Henning et al. (2019) [38]. After simulated vibration emission treatment, semen was stained using Hoechst 33342 (Beyotime Institute of Biotechnology, Shanghai, China) at a final concentration of 1.25 mg/mL and incubated for 30 min at room temperature. Healthy sows’ oviducts were obtained from abattoirs and transported to the laboratory in cold phosphate-buffered saline. Tissue segments (explants) of 0.5–1.0 mm in length were cut from the longitudinal folds of the oviductal isthmic and stored in Tyrode’s medium. Only explants exhibiting rapid ciliary beating along the intact explant ridges were utilized. A total of 18 explants were prepared, sourced from two distinct sows, with each contributing nine explants. Explants from each sow were allocated to different wells of a 24-well plate containing 490 µL pre-warmed and equilibrated Tyrode’s medium (38 °C; 5% CO_2_, 100% humidity). An equal amount of stained sperm (2 × 10^5^ sperm) was added to each well, followed by a 45 min co-incubation in a CO_2_ incubator (38 °C; 5% CO_2_, 100% humidity). Subsequently, explants were transferred to fresh, pre-warmed, and equilibrated Tyrode’s medium, gently washed to remove loosely bound sperm, and placed onto glass slides containing 60 µL of Tyrode’s medium, surrounded by a silicone grease frame and sealed with a coverslip. Three sites of dense sperm binding on the intact explant surface were assessed to avoid edge effects from explant cutting. Using fluorescence microscopy, all layers of the explants at the same positions were consecutively focused, and images from different focal planes were merged. The explant area was measured, and a sperm count was conducted.

The average number of bound sperm per square millimeter was defined as the BI. According to the description of Henning et al. (2022) [39], the BI calculation formula is as follows (Equation (2)):

(2) BI=(NI1+NI2+NI3)/(AI1+AI2+AI3)

where

Al1, Al2, Al3 = area of location no. 1, 2, and 3, respectively;
Nl1, Nl2, Nl3 = the number of sperm at location no. 1, 2, and 3, respectively.

### 2.18. Insemination Assays

Employing the back pressure, we simultaneously examined and validated the estrus of a collective total of 50 sows. The sows were randomly assigned to control and experimental groups. Within 12 and 24 h after estrus detection, two cervical AI were performed on each sow, using 80 mL of semen containing 2.4 × 10^9^ sperm per dose. The semen doses used for two successive cervical AI are sourced from the same batch of semen that was divided from the mixed semen from 15 boars’ ejaculates. Twenty-five sows were inseminated with the semen treated with simulated vibration emissions for 3 h. The other sows were inseminated with the untreated semen storage at 17 °C as a control. All inseminations were conducted by trained technicians. Between 18 and 24 days after insemination, the return to estrous was assessed. Early abortions were monitored using ultrasound (GDF-C70, Gandaofu, Zhengzhou, China). Pregnancy was confirmed by ultrasound 28 days after inseminations. The farm personnel recorded the total born litter size, live-born litter size, and healthy born litter size.

### 2.19. Statistical Analysis

All statistical analyses were performed using IBM SPSS Statistics 24 (IBM Corp., Armonk, NY, USA). All the data were tested for normality and variance homogeneity prior to statistical analysis. The data were transformed by arcsine square root transformation when necessary. To analyze the effects of different processing times and rotational speeds on sperm motility parameters, acrosome integrity, and plasma membrane integrity, and for comparisons between groups treated at 200 rpm for different durations, we employed one-way ANOVA on the repeated measurement data, followed by Tukey’s post hoc test. We calculated the standard deviation and coefficient of variation of the obtained vibration data to assess the dispersion and relative variability levels within the categorized vibration intensities. To demonstrate the differences in reproductive parameters between the two groups in response to vibration, we conducted pairwise comparisons using independent samples *t*-tests. Values are considered to be statistically significant when *p* < 0.05.

## 3. Results

### 3.1. Analysis of Vibration Emissions under Field Conditions and Orbital Shaker

Throughout the entire 1500 km journey, the average displacement ($D_{\bar{x}}$) was 0.26 (t = 53.3 h). To analyze the relative frequency of different vibration intensities occurring throughout the entire journey, we have categorized and summarized data based on varying displacement levels. Here, we observe that displacement levels are predominantly distributed below 0.2, accounting for 51.37%, with the 0.06~0.12 range representing 22.54%. The specific summary data are provided in Table 1 and Table S1. Despite over 80% of displacement levels being below 0.5 under normal transportation conditions, it was still unavoidable that isolated events exhibited higher vibrations (10.01%) and strong vibrations (1.62%).

To investigate the relationship between road vibration emissions and laboratory conditions, we utilized an orbital shaker to simulate vibrational emissions. By comparing the D values under road and experimental conditions, we observed that at 80, 140, and 200 rpm on the orbital shaker, the median D values were within the ranges of 0.1~0.2, 0.5~1.0, and 1.0~2.0 under road conditions, respectively (Table 2). The median values of the sensor displacement index exhibited noticeable differences (*p* < 0.05), increasing with the rise in rotational speed (Figure S1). Therefore, under laboratory conditions, we chose 80, 140, and 200 rpm to simulate low, high, and strong vibrations for the vibrational emission treatment of sperm.

### 3.2. Effect of Vibration Emissions on Sperm Motility Parameters

The results of the sperm motility data under various rotational speeds (0, 80, 140, 200 rpm) after 3 and 6 h are presented in Table 3. There was no significant difference in sperm motility parameters between 0 h samples without vibration treatment (*p* > 0.05). Compared to the control group, the vibration emission treatment significantly reduced sperm total motility (*p* < 0.05). The poorest total motility was observed in samples treated for 6 h at 200 rpm. Compared to the control group, vibration emission treatment also significantly decreased sperm progressive motility (*p* < 0.05), with similar progressive motility observed in samples treated at 80 rpm and 140 rpm. The poorest progressive motility was observed in samples treated for 6 h at 200 rpm. Total motility was decreased on average by between 8.8 and 26.2% after vibration emission treatment compared to the controls, and progressive motility was reduced on average by between 7.5 and 23.4% after vibration emission treatment. Following vibration emission treatment for 3 h and 6 h, sperm parameters including VCL, VSL, VAP, LIN, STR, ALH, and BCF were decreased. Sperm samples treated at 80 rpm and 140 rpm exhibited similar values for VCL, VAP, STR, WOB, ALH, and BCF.

The sperm motility data results after processing sperm at 200 rpm for 3 and 6 h and storage at 17 °C for up to 96 h are presented in Table 4 and Table S2. During the storage at 17 °C up to 96 h, the values of total motility, progressive motility, as well as parameters including VCL, VSL, VAP, LIN, and WOB in sperm treated with vibration emissions were significantly reduced (*p* < 0.05). Interestingly, samples treated for 6 h at 200 rpm exhibited the poorest sperm total motility and progressive motility at each point of storage at 24, 48, 72, and 96 h among the treatments (*p* < 0.05).

### 3.3. Effects of Vibration Emission Stress on Sperm Plasma Membrane Integrity, Acrosome Integrity, Abnormality Rate, and pH Value

**Figure 1:**
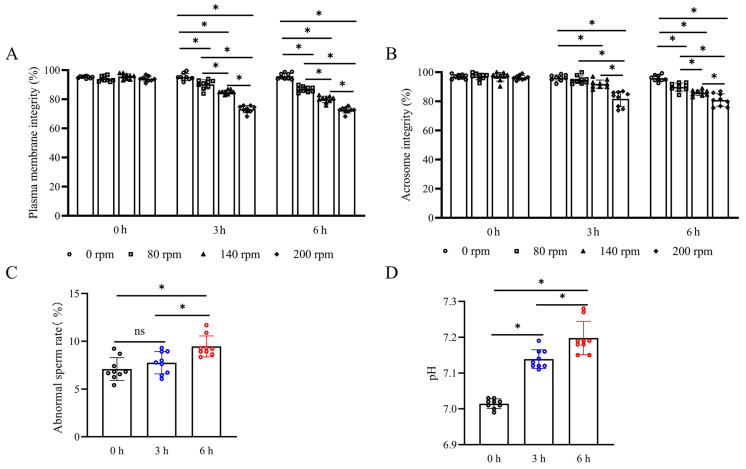
Detection of plasma membrane integrity, acrosome integrity, abnormality rate, and pH value after treatment with vibration emissions; n = 9. Simulated transport-associated vibration was achieved by shaking 80 mL of semen at a speed of 200 rpm at time points of 0, 3, and 6 h. (**A**) Effects of vibration emissions on boar sperm plasma membrane integrity after 3 and 6 h of treatments at different rotational speeds. (**B**) Effects of vibration emissions on boar sperm acrosome integrity after 3 and 6 h of treatments at different rotational speeds. (**C**) Changes in sperm abnormality rates at different time points under simulated vibration emission treatment. (**D**) Changes in semen pH values at different time points under simulated vibration emission treatment. Values are specified as mean ± standard deviation. Significant differences between groups, * *p* < 0.05. ns means not significantly between groups.

As shown in Figure 1A,B, both sperm plasma membrane integrity and acrosome integrity were decreased when sperm was under the stress of vibration emissions. Notably, the treatment at a rotational speed of 200 rpm had the lowest percentage of sperm plasma membrane integrity and acrosome integrity (at 3 h and 6 h point, plasma membrane integrity and acrosome integrity are 73.17 ± 2.44% and 72.54 ± 1.98% and 81.72 ± 5.23% and 80.86 ± 4.33%, respectively). There was no significant difference in the simulated vibration emission time extension at this speed (*p* > 0.05). Smaller vibration frequencies did not show a significant difference in damage to sperm acrosome integrity over a short duration (Figure 1B) (*p* < 0.05). At the 3 h point, sperm acrosome integrity decreased by 2.29% and 5.02% in treatments at 80 rpm and 140 rpm, respectively. However, as the duration was extended, the vibration frequency significantly decreased sperm acrosome integrity compared to the control group (*p* < 0.05). Vibrational stress significantly impacts both the sperm abnormality rate and semen pH values. As shown in Figure 1C,D, semen pH demonstrated a significant increase following vibrational exposure at 3 and 6 h compared to the control group (7.14 and 7.20, respectively) (*p* < 0.05). The sperm abnormality rate was 8.12 ± 0.84% at 3 h post-vibration exposure (*p* > 0.05) and increased to 9.45 ± 1.04% at 6 h post-exposure (*p* < 0.05).

### 3.4. Effects of Simulated Vibration Emissions on Sperm Tyrosine Phosphorylation Levels, ATP Levels, and Mitochondrial Membrane Potential

**Figure 2:**
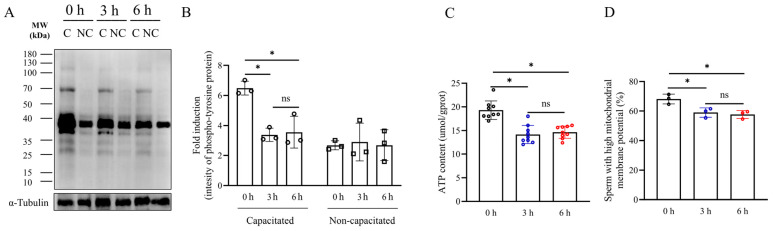
Effects of vibration emissions on sperm mitochondrial membrane potential, ATP levels, and tyrosine phosphorylation levels. Simulated transport-associated vibration was achieved by shaking 80 milliliters of semen at a speed of 200 rpm at time points of 0, 3, and 6 h. (**A**) Western blotting images showing the expression of Phospho-Tyrosine protein and α-tubulin in boar sperm. (**B**) Quantitative expression of Phospho-Tyrosine generated by western blotting relative to α-tubulin (control), n = 3. (**C**) Changes in ATP levels in boar sperm at different times of simulated vibration emission treatment, n = 9. (**D**) Effects of different lengths of vibration emission treatment on the boar sperm mitochondrial membrane potential, n = 3. Values are specified as mean ± standard deviation of three replicates. Significant differences between groups, * *p* < 0.05. ns means not significantly between groups. C: capacitated sperm sample; NC: non-capacitated sperm sample.

To study the effect of simulated vibration emissions on the capacitation of boar sperm, the tyrosine phosphorylation levels of boar sperm were detected by western blot (Figure 2A,B and Supplementary Figure S5). The results revealed a significant reduction in the tyrosine phosphorylation level of boar sperm following exposure to vibration emissions compared to the control group (Figure 2A,B and Supplementary Figure S5) (*p* < 0.05). Notably, no significant disparity in tyrosine phosphorylation levels was observed between the 3 h and 6 h treatment groups (Figure 2B) (*p* > 0.05). The sperm ATP levels were significantly decreased at a rotational speed of 200 rpm after both 3 and 6 h of treatment (Figure 2C) (*p* < 0.05). The mitochondrial membrane potential of sperm samples was significantly decreased at a rotational speed of 200 rpm after both 3 and 6 h of treatment (Figure 2D and Supplementary Figure S6) (*p* < 0.05).

### 3.5. Effects of Vibration Emissions on the Expression of Mitochondria-Encoded Proteins and the Levels of Mitochondrial ROS

**Figure 3:**
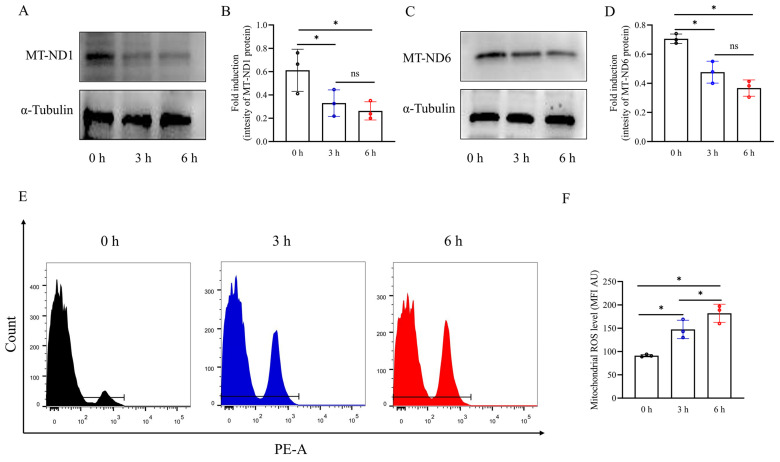
Western blotting images showing the levels of mitochondria-encoded proteins (MT-ND1, MT-ND6) and α-tubulin. (**A**,**C**) Western blotting image showing the expression of the MT-ND1 and MT-ND6, and α-tubulin. (**B**,**D**) Quantitative expression of MT-ND 1 and MT-ND 6 produced by western blotting relative to α-tubulin (control). (**E**,**F**) Mitochondrial ROS levels in sperm after vibration emission treatment for 0, 3, and 6 h, with MFI analysis conducted on positively stained sperm. Higher MFI values indicate elevated ROS levels in sperm. MFI: mean fluorescence intensity; AU: arbitrary units; n = 3. Simulated transport-associated vibration was achieved by shaking 80 milliliters of semen at a speed of 200 rpm at time points of 0, 3, and 6 h. Values are specified as mean ± standard deviation of three replicates. Significant differences between groups, * *p* < 0.05. ns means not significantly between groups.

To investigate the effect of vibration emissions on the expression of mitochondrial-encoded proteins in boar sperm, western blot was used to detect the levels of mitochondrial-encoded proteins in boar sperm. As shown in Figure 3A–D and Supplementary Figures S3 and S4, it was observed that the vibration emission treatment significantly reduced the total levels of MT-ND1 and MT-ND6 (*p* < 0.05). Vibration emissions significantly increase mitochondrial ROS in a time-dependent manner (Figure 3E,F). The highest levels were observed at 6 h (Figure 3F) (*p* < 0.05).

### 3.6. Effects of Simulated Vibration Emissions on Sperm Lactate Levels, HK Activity, PK Activity, and LDH Activity

**Figure 4:**
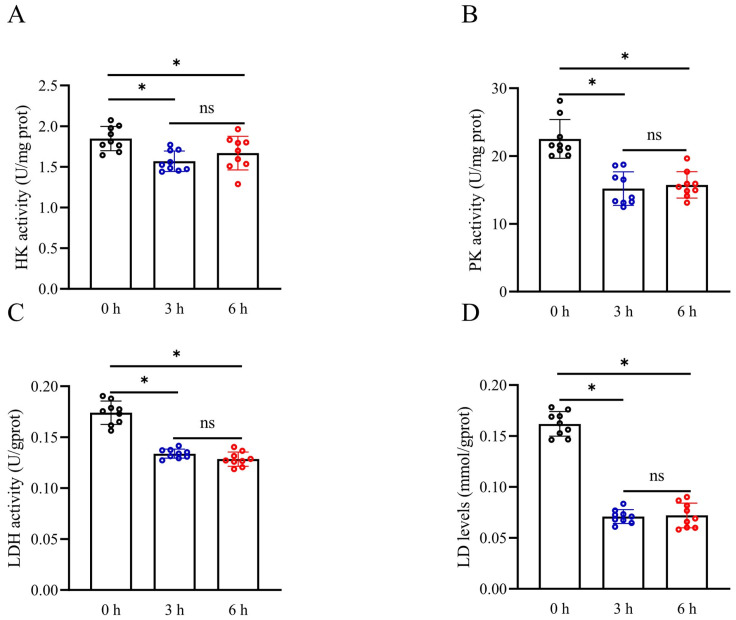
Effects of vibration emissions on boar sperm HK activity (**A**), PK activity (**B**), LDH activity (**C**), and lactate levels (**D**); n = 9. Simulated transport-associated vibration was achieved by shaking 80 milliliters of semen at a speed of 200 rpm at time points of 0, 3, and 6 h. Values are specified as mean ± standard deviation. Significant differences between groups, * *p* < 0.05. ns means not significantly between groups. HK: hexokinase; PK: pyruvate kinas; LDH: lactate dehydrogenase.

Lactate levels, HK activity, PK activity, and LDH activity of sperm were measured after simulated vibration emissions to assess the effect of vibration emissions on sperm glycolytic metabolism. The activities of HK, PK, and LDH in sperm were observed to be significantly decreased after 3 and 6 h of simulated vibration emissions compared to the control group (Figure 4A–C) (*p* < 0.05). Interestingly, prolonging the duration of simulated vibration emissions up to 6 h did not significantly reduce HK, PK, and LDH activities compared to the 3 h exposure (*p* > 0.05). Moreover, the levels of lactate, a byproduct of glycolysis, were also observed to decrease following exposure to simulated vibration emissions (Figure 4D) (*p* < 0.05).

### 3.7. Effects of Simulated Vibration Emissions on Sperm Binding to Oviduct Explants

**Figure 5:**
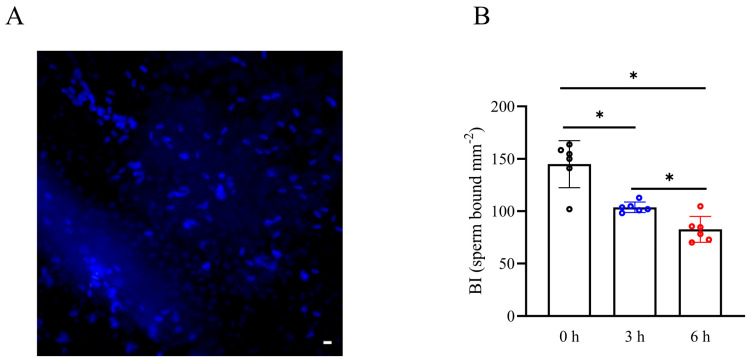
Effects of vibration emissions on boar sperm binding in the oviduct explant. An oviduct explant assay was applied to compare the BI for semen samples after vibration emission treatment. (**A**) Illustrative image of Hoechst 33342-tagged sperm bound to the oviduct explant surface (scale bar = 60 µm). (**B**) Effects of vibration emission treatment on sperm BI; n = 6. Simulated transport-associated vibration was achieved by shaking 80 milliliters of semen at a speed of 200 rpm at time points of 0, 3, and 6 h. Values are specified as mean ± standard deviation. Significant differences between groups, * *p* < 0.05.

To investigate the impact of simulated vibration radiation on the fertilization capacity of boar sperm, we utilized Hoechst 33342 dye for sperm staining and assessed the sperm’s ability to form a “reservoir” in the oviduct through the sperm BI. As shown in Figure 5A,B, the sperm BI in 200 rpm vibration emission treatment was lower than that of the control. It was also observed that the reduction in the sperm BI in 200 rpm vibration emission stress occurred in a time-dependent manner, and it showed the lowest level after 6 h of 200 rpm vibration emissions (Figure 5B) (*p* < 0.05). This suggested that vibration emissions impair the ability of sperm to form a reservoir.

### 3.8. Effects of Simulated Vibration Emissions on Reproductive Parameters

**Figure 6:**
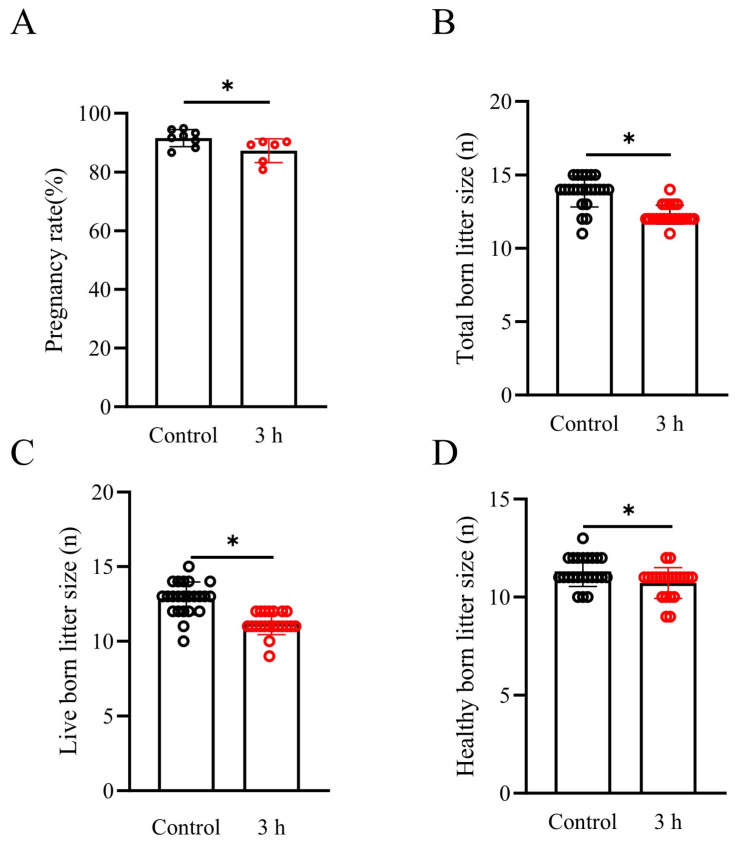
Effects of simulated vibration emissions on reproductive parameters; n = 25. Simulated transport-associated vibration was achieved by shaking 80 milliliters of semen at a speed of 200 rpm at time points of 0 and 3 h. Effects of vibrational emissions on the pregnancy rate (**A**), total born litter size (**B**), live-born litter size (**C**), and healthy born litter size (**D**) in sows. Values are specified as mean ± standard deviation. Significant differences between groups, * *p* < 0.05.

As the sperm BI in 6 h vibration emission treatment was much lower than that in the 3 h vibration emission treatment or the control group, we considered the loss of economic benefits resulting from the AI that suffered from vibration emission stress. In this study, we only performed AI in sows using both untreated boar sperm and boar sperm treated with 3 h vibration emissions to evaluate the reproductive parameters. The control semen dose was kept at 17 °C for 3 h without vibration emissions, and artificial insemination was carried out simultaneously with the pig sperm after vibration emissions. As shown in Figure 6A–D, the results indicate that when sperm was exposed to vibration emissions, compared to the control group (with pregnancy rate, total born litter size, live-born litter size, and healthy born litter size being 91.54 ± 2.89%, 13.86 ± 1.06, 12.87 ± 1.10, and 11.30 ± 0.76, respectively), there was a significant reduction in sow pregnancy rate (87.27 ± 4.06%), total born litter size (12.28 ± 0.64), live-born litter size (11.19 ± 0.75), and healthy born litter size (10.71 ± 0.78) (*p* < 0.05). This suggests that the vibration emission treatment has a significant impact on the reproductive parameters in sows.

## 4. Discussion

In modern pig-breeding systems, reducing the loss of boar sperm quality during transportation is an effective way to improve the efficiency of AI [3]. Due to the concentration of semen production units and the considerable distance between boar studs and sow farms, in China, the typical transport times for most semen doses range from 10 to 20 h, with some being transported for more than 40 h (Figure S2). In this study, it was observed that the quality of boar sperm, particularly total motility, progressive motility, plasma membrane integrity, and acrosome integrity, significantly deteriorated following exposure to vibration emissions. Furthermore, when the vibration frequency was excessively high, it impaired sperm quality, overshadowing the influence of prolonged transportation time on sperm, especially within a short timeframe. In this study, vibration emissions led to an alkalization of semen pH, which increased with the prolonged exposure to vibration emissions. The elevation in pH may impact intracellular pH, thereby influencing sperm metabolism and compromising sperm vitality [40,41]. Previous research suggests that even in the absence of significant pH changes, sperm motility still decreases [4]. This suggests that pH variation might not be the primary mechanism behind the decline in sperm motility. Furthermore, when using sperm subjected to vibration emission treatment for artificial insemination, sow pregnancy rates, total born litter size, live-born litter size, and healthy born litter size were significantly lower than those in the control group. It was observed that vibration emissions affected sperm morphology, although changes were not apparent in the short term; significant differences in sperm morphology became evident with extended exposure duration. Sperm morphology abnormalities are typically associated with lower fertilization capacity and potential DNA abnormalities [42]. However, in this study, changes in sperm morphology were induced by external forces, and the sperm abnormality rate remained at relatively low levels (8.12 ± 0.84% at 3 h and 9.45 ± 1.04% at 6 h). Previous studies indicate that abnormalities in sperm morphology do not affect the pregnancy rate and live birth rate in AI [43]. However, a significant correlation has been observed with the number of offspring born [44]. In the present study, after 3 h of vibration emission treatment, there was no significant difference observed in the sperm abnormality rate compared to the control group. Therefore, the observed reductions in pregnancy rate, total born litter size, live-born litter size, and healthy born litter size in this study may not be related to changes in sperm morphology. Adequate motility is the key to successful fertilization [36], as boar sperm with poor morphology and motility are filtered out by cervical mucus [45]. The acrosome and membrane of the sperm have a synergistic effect and play an essential role in fertilization [46], where abnormal acrosome reactions reduce male fertility [47]. In the present study, vibration emissions presented a negative effect on sperm viability parameters, and these findings are consistent with those of Schulze et al. (2018) [8] and Paschoal et al. (2021) [4], who reported that vibration emissions altered extender pH, increased sperm ROS levels, and induced shear stress. Additionally, impaired sperm viability was associated with reduced fertility [48]. Therefore, minimizing the damage caused by vibration emissions is crucial for maintaining sperm viability during AI dose transportation.

Sperm are specialized, terminally differentiated cells that rely on both glycolysis and the mitochondrial oxidative phosphorylation (OXPHOS) pathways to generate ATP, which is essential for sustaining their functions [49]. Sperm viability is intricately linked to ATP production [36]. Within sperm, a significant concentration of glycolytic enzymes is found in the fibrous sheath, enabling a high throughput of ATP production locally [50]. In a mouse model, the inhibition of sperm glycolysis led to a dramatic reduction in flagellar beating and overall motility [51]. Additionally, lactate, a byproduct of sperm glycolysis, can further serve as a source of mitochondrial energy by re-oxidizing to pyruvate in both the mitochondrial matrix and cytosol [52,53]. This study revealed that the activities of hexokinase (HK), pyruvate kinase (PK), and lactate dehydrogenase (LDH), as well as the levels of lactate, were significantly lower in sperm exposed to vibration emissions compared to the control group, suggesting that vibration emissions hinder ATP production in boar sperm by impeding glycolysis. Therefore, reducing vibration amplitudes or shortening transport times may prove beneficial in preserving sperm glycolytic metabolism, thus maintaining sperm motility during AI dose transportation.

Mature mammalian sperm possess mitochondria with a highly concentrated structure and a compact matrix, enhancing the efficiency of energy production [23]. ATP generated from mitochondria plays a crucial role in sperm fertilization and capacitation [54,55]. When mitochondrial ATP synthesis is diminished, both sperm motility and in vitro sperm capacitation are inhibited [56]. Mitochondrial membrane potential reflects the proton gradient formed during the passage of electrons through the electron transport chain [23], and a reduction in membrane potential signifies mitochondrial damage [57]. Previous studies have shown a relationship between mitochondrial activity and electron transport in the respiratory chain [48], and proteins involved in the mitochondrial electron transport chain are crucial for maintaining mitochondrial activity [58]. Moreover, in a prior study, increased expression and synthesis of mitochondrial genes and proteins were found to enhance mitochondrial activity and ATP levels, leading to improved sperm linear movement [59]. ROS were byproducts of the mitochondrial ATP generation process [60], reducing ATP production by influencing mitochondrial activity [61]. In this study, following vibrational emission treatment, there was an elevation in ROS levels within sperm mitochondria. This elevation was accompanied by a significant reduction in sperm mitochondrial membrane potential, ATP levels, and the levels of mitochondrial-encoded proteins (MT-ND1 and MT-ND6). Through a comparison of sperm with different fertilities, research has revealed that proteins involved in oxidative phosphorylation and pyruvate metabolism pathways are more abundant in highly fertile sperm [62]. It is widely recognized that cellular proteins may undergo degradation and lose functionality due to ROS-induced damage [63]. Additionally, Complex I and Complex III have been identified as two primary sites for ROS generation within the cellular electron transport chain (ETC) under both normal metabolic conditions and stress-induced conditions [26]. Given that MT-ND1 and MT-ND6 are subunits of Complex I, located proximally to the ROS generation site [64], these ETC proteins are likely to be particularly susceptible to severe oxidative damage caused by elevated ROS exposure. The damage to electron transport chain (ETC) proteins may be a factor influencing reproductive parameters. In the present study, exposure to vibration emission stress resulted in a significant reduction in sperm mitochondrial membrane potential, levels of mitochondrial-encoded proteins (MT-ND1 and MT-ND6), and ATP levels. These findings indicate that vibration emissions may impair the function of boar sperm mitochondria, leading to reduced ATP production through the OXPHOS pathway. Consequently, during AI dose transportation, safeguarding sperm mitochondrial function by minimizing vibration emission stress is crucial for maintaining boar sperm motility.

Sperm capacitation is a crucial step for sperm to successfully fertilize an oocyte [65]. During the capacitation process, protein tyrosine phosphorylation levels typically increase [66]. In this study, a significant reduction in sperm tyrosine phosphorylation levels was observed following exposure to vibration emissions. Lower levels of tyrosine phosphorylation indicate a decrease in fertilization potential [66]. The binding of sperm to the epithelial cells of the oviduct, establishing a sperm reservoir, represents another critical step in successful fertilization. Only viable, non-capacitated, and functionally intact sperm can bind to oviductal epithelial cells [67]. Assessing sperm binding to oviductal explants in vitro can reflect the relative fertility of males [68]. This study found that exposure to vibration emissions significantly reduced the sperm binding index, indicating that vibration emissions impair the ability of sperm to form a reservoir within the female reproductive tract, thereby hindering the development of pig breeding. As previously stated, following vibration emission treatment, there was a significant decrease in the quality and fertilization capability of sperm, accompanied by impairment of glycolytic pathways and mitochondrial function. When these sperm are utilized for AI, the sow’s pregnancy rate, total born litter size, live-born litter size, and healthy born litter size are markedly lower than those of the control group. Hyperactive motility and tyrosine phosphorylation serve as two indicators of sperm capacitation [69]. Hyperactive motility is observable only when glycolysis is unimpeded [28], and the integrity of mitochondrial function is an indispensable aspect of the capacitation process. Therefore, vibration emissions may potentially impact sperm capacitation by inhibiting the glycolytic pathway and influencing mitochondrial function, ultimately affecting reproductive capability.

High-quality sperm is crucial for successful fertilization and healthy embryo development. Implementing protective measures ensures that sperm morphology, motility, and functionality are optimized, thereby increasing the chances of successful conception. We recommend devising relevant logistics and transportation strategies to minimize the impact of vibrations during the transportation of liquid-stored semen. Optimal logistics strategies should be chosen to minimize transportation time and reduce air content in semen tubes. Upgrading and optimizing semen transportation packaging methods to minimize vibration emissions, selecting appropriate sperm extenders, and establishing a robust quality assurance system for semen transportation are advised. Real-time monitoring of parameters affecting sperm quality during transportation, the choice of different types of transportation vehicles and shock absorbers, and planning of the best transportation routes are essential components of this system. Implementing these measures may contribute to maintaining semen quality during the transportation process.

## 5. Conclusions

**Figure 7:**
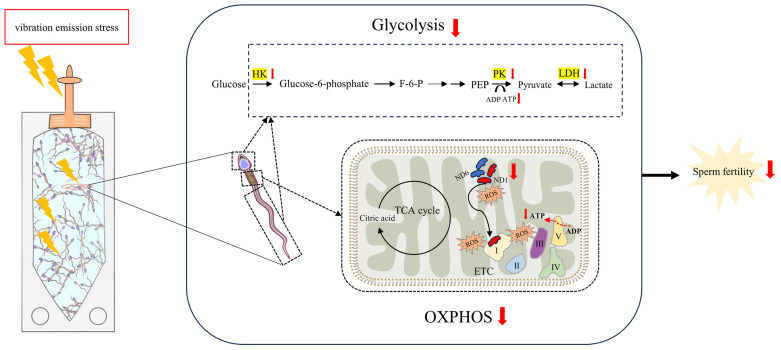
Mechanisms of damage to sperm function by vibration emissions induced with transportation. When boar sperm are subjected to the stress of vibration emissions, there is an induced increase in reactive oxygen species (ROS) produced by mitochondria. Excessive ROS attack the mitochondria, leading to damage to mitochondrial proteins such as MT-ND1 and MT-ND6. Additionally, vibration radiation suppresses the activity of glycolytic enzymes in the head and tail regions of boar sperm, resulting in a downregulation of both glycolysis and OXPHOS pathways. Consequently, this cascade of effects reduces ATP production, ultimately compromising the reproductive capability of boar sperm. F-6-P: fructose 6-phosphate. PEP: phosphoenolpyruvate. OXPHOS: oxidative phosphorylation. ETC: electron transport chain. TCA: tricarboxylic acid. HK: hexokinase. PK: pyruvate kinase. LDH: lactate dehydrogenase.

In conclusion, as illustrated in Figure 7, the vibration emissions experienced by AI doses during the transport process induce an increase in ROS generated by mitochondria. Concurrently, there is a downregulation of the activity of glycolytic pathway enzymes and the levels of mitochondrial-encoded proteins (MT-ND1 and MT-ND6) in sperm. This results in the inactivation of glycolysis and oxidative phosphorylation pathways in boar sperm, leading to a reduction in ATP levels and ultimately compromising sperm fertilization capability. The present study provides new insights into understanding how the vibration emissions damage sperm fertilization function during transportation, contributing to improvements in AI.

## Figures and Tables

**Table 1:** By employing vibration sensors to measure the occurrence of vibrations during the actual transportation process, we utilized the spatial displacement changes between two consecutive time points to represent the intensity of the vibrations.

Range (D)	N	Median (D)	SD (D)	CV (D)
0.0~0.1	24,270	0.07	0.02	0.34
0.1~0.2	25,064	0.14	0.03	0.2
0.2~0.3	15,491	0.25	0.03	0.12
0.3~0.5	15,293	0.37	0.06	0.15
0.5~1.0	14,698	0.64	0.13	0.2
1.0~2.0	1212	1.15	0.21	0.17

D = spatial displacement changes between two consecutive time points; N = frequency of occurrences within the transportation time interval. Median values, standard deviations (SD), and coefficient of variation (CV) across various ranges of vibration.

**Table 2:** By employing vibration sensors to measure the occurrence of vibrations in the laboratory orbital shaker, we utilized the spatial displacement changes between two consecutive time points to represent the intensity of the vibrations.

Shaker Speed (rpm)	N	Median (D)	SD (D)	CV (D)
80	1200	0.11	0.09	0.67
140	1200	0.55	0.6	0.82
200	1200	1.55	1.5	0.79

D = spatial displacement changes between two consecutive time points; N = frequency of occurrences within the transportation time interval. Median values, standard deviations (SD), and coefficient of variation (CV) across various ranges of vibration.

**Table 3:** Effect of different rotational speeds (80, 140, and 200 rpm) and treatment times (0, 3, and 6 h) on boar sperm motility parameters determined by CASA; n = 9.

	Time (h)	Shaker Speed (rpm)			
Parameter		0 rpm	80 rpm	140 rpm	200 rpm
Total motility (%)	0	95.3 ± 0.8	95.7 ± 0.4	93.6 ± 1.9	93.0 ± 2.1
	3	94.9 ± 0.4 ^a^	86.9 ± 0.6 ^b^	79.6 ± 1.8 ^c^	67.9 ± 1.5 ^d^
	6	93.6 ± 1.8 ^a^	81.4 ± 0.5 ^b^	74.0 ± 1.6 ^c^	66.8 ± 2.8 ^d^
Progressive motility (%)	0	71.1 ± 2.7	70.1 ± 5.7	70.2 ± 4.6	69.5 ± 4.9
	3	70.0 ± 2.2 ^a^	62.6 ± 1.6 ^b^	61.4 ± 0.2 ^b^	53.1 ± 1.1 ^c^
	6	71.4 ± 3.1 ^a^	58.8 ± 1.1 ^b^	57.1 ± 0.8 ^b^	46.1 ± 3.4 ^c^
VCL (μm/s)	0	99.6 ± 1.7	99.6 ± 3.2	99.2 ± 3.2	98.4 ± 3.3
	3	99.2 ± 1.8 ^a^	95.8 ± 3.5 ^a^	89.6 ± 6.1 ^a^	64.1 ± 7.9 ^b^
	6	99.1 ± 0.3 ^a^	91.9 ± 7.4 ^a^	85.4 ± 12.4 ^a^	61.4 ± 3.3 ^b^
VSL (μm/s)	0	33.5 ± 0.9	33.6 ± 4.2	33.9 ± 3.9	32.9 ± 3.8
	3	34.0 ± 1.2 ^a^	32.5 ± 3.6 ^a^	27.6 ± 0.4 ^b^	20.6 ± 1.1 ^c^
	6	33.9 ± 1.8 ^a^	29.7 ± 1.6 ^b^	23.8 ± 1.6 ^c^	18.3 ± 1.7 ^d^
VAP (μm/s)	0	51.5 ± 0.8	52.1 ± 7.8	52.3 ± 7.4	51.3 ± 7.3
	3	52.9 ± 1.6 ^a^	47.8 ± 1.7 ^b^	42.5 ± 1.3 ^c^	36.7 ± 1.6 ^d^
	6	52.6 ± 2.0 ^a^	42.6 ± 0.9 ^b^	40.5 ± 2.0 ^b^	33.7 ± 0.9 ^c^
LIN (%)	0	48.3 ± 1.7	49.1 ± 2.8	48.8 ± 3.0	48.0 ± 2.5
	3	47.8 ± 1.9 ^a^	36.4 ± 0.7 ^b^	32.0 ± 1.4 ^c^	30.5 ± 0.7 ^c^
	6	48.4 ± 2.1 ^a^	33.5 ± 3.0 ^b^	29.1 ± 1.5 ^c^	29.7 ± 1 ^bc^
STR (%)	0	73.9 ± 4.0	74.9 ± 3.6	73.8 ± 3.4	73.2 ± 3.7
	3	73.9 ± 3.7 ^a^	69.3 ± 2.2 ^ab^	64.3 ± 0.8 ^b^	55.9 ± 3.5 ^c^
	6	73.1 ± 0.7 ^a^	67.5 ± 2.3 ^b^	60.7 ± 2.8 ^c^	55.9 ± 2.1 ^d^
WOB (%)	0	65.3 ± 0.8	65.8 ± 1.0	66.2 ± 1.3	65.0 ± 1.5
	3	65.5 ± 0.5 ^a^	49.7 ± 1.2 ^c^	49.9 ± 3.5 ^c^	54.6 ± 2.5 ^b^
	6	65.5 ± 0.3 ^a^	48.2 ± 2.2 ^c^	48.3 ± 0.8 ^c^	53.6 ± 1.2 ^b^
ALH (μm)	0	6.7 ± 0.4	6.8 ± 0.2	6.7 ± 0.2	6.6 ± 0.3
	3	6.7 ± 0.1 ^a^	6.0 ± 0.4 ^b^	5.7 ± 0.3 ^b^	3.7 ± 0.3 ^c^
	6	6.8 ± 0.1 ^a^	5.7 ± 0.8 ^a^	5.5 ± 0.9 ^a^	3.3 ± 0.4 ^b^
BCF (Hz)	0	5.9 ± 0.1	5.9 ± 0.2	5.8 ± 0.1	5.8 ± 0.1
	3	5.8 ± 0.3 ^a^	5.3 ± 0.2 ^b^	5.1 ± 0.2 ^b^	4.4 ± 0.1 ^c^
	6	5.8 ± 0.3 ^a^	4.7 ± 0.1 ^b^	4.5 ± 0.1 ^b^	4.1 ± 0.1 ^c^

Values are expressed as mean ± standard deviation. Different letters within a row indicate significant difference (*p* < 0.05). VCL, curvilinear velocity; VSL, straight-line velocity; VAP, average path velocity; BCF, beat-cross frequency; ALH, lateral head; STR, straightness (VSL/VAP); LIN, linearity (VSL/VCL); WOB, wobble (VAP/VCL). ^a–c^ Different letters within the same low are significantly different (*p* < 0.05).

**Table 4:** With a rotational speed of 200 rpm, sperm were processed for different durations (0, 3, and 6 h). Subsequently, during the storage of the semen (24, 48, 72, and 96 h), the influence of vibrations on sperm motility was assessed through CASA; n = 9.

Parameter	Time (h)	0 h	24 h	48 h	72 h	96 h
Total motility (%)	0	96.2 ± 2.8 ^ab^	98.9 ± 0.6 ^a^	95.1 ± 1.4 ^bc^	92.5 ± 1.8 ^cd^	90.1 ± 2.5 ^d^
	3	93.2 ± 5.1 ^a^	78 ± 1.9 ^b^	70.7 ± 4.8 ^c^	69.5 ± 3.0 ^c^	73.6 ± 2.7 ^bc^
	6	96.2 ± 1.9 ^a^	66.6 ± 3.2 ^b^	69.8 ± 4.6 ^b^	64.4 ± 3.5 ^b^	67.3 ± 0.5 ^b^
Progressive motility (%)	0	72.2 ± 2.3 ^a^	71.1 ± 6.5 ^a^	70.3 ± 7.6 ^ab^	65.9 ± 2.5 ^ab^	60.9 ± 2.5 ^b^
	3	71.2 ± 1.7 ^a^	52.7 ± 3.4 ^b^	44.1 ± 9.9 ^b^	40.7 ± 5.9 ^b^	40.6 ± 1.3 ^b^
	6	71.8 ± 1.7 ^a^	50.0 ± 4.6 ^b^	39.8 ± 3.8 ^c^	32 ± 2.2 ^d^	29.6 ± 2.4 ^d^

Values are expressed as mean ± standard deviation. ^a–d^ Different letters within the same low are significantly different (*p* < 0.05).

## Data Availability

The data presented in this study are available in the article.

## Supplementary Materials

The following supporting information can be downloaded at: https://www.mdpi.com/article/10.3390/biology13060370/s1, Figure S1: The displacement levels corresponding to different rotational speeds; Figure S2: Semen delivery data from five boar stations in various regions of China between 2022 and 2023; Figure S3: The original western blotting images showing the levels of mitochondria-encoded proteins MT-ND1 and α-Tubulin; Figure S4: The original western blotting images showing the levels of mitochondria-encoded proteins MT-ND6 and α-Tubulin; Figure S5: Effects of vibration emissions on sperm tyrosine phosphorylation level and α-Tubulin; Figure S6: Effects of vibration emissions on sperm mitochondrial membrane potential. Table S1: During the transportation process, the ratio of spatial displacement within different ranges to the entire transportation process; Table S2: Effect of the vibration emissions on sperm motility parameters.
