# Supplementary material for: Vibration Emissions Reduce Boar Sperm Quality via Disrupting Its Metabolism
Source: Biology (Basel). 2024 May 23;13(6):370. doi: 10.3390/biology13060370 (PMC11200616; doi:10.3390/biology13060370)
Supplement: Supplementary file 1 [file biology-13-00370-s001.zip › biology-2915796-supplementary.pdf]

Supplementary

Table S1. During the transportation process, the ratio of spatial displacement within different ranges to the entire transportation process. By employing vibration sensors to measure the occurrence of vibrations during the actual transportation process, we utilize the spatial displacement changes between two consecutive time points to represent the intensity of the vibrations.

Range (D)	N	Percentage
0.00 to 0.06	10107	11.16%
0.06 to 0.12	20418	22.54%
0.12 to 0.18	14996	16.55%
0.18 to 0.24	10587	11.69%
0.24 to 0.30	8717	9.62%
0.30 to 0.36	6372	7.03%
0.36 to 0.42	4743	5.24%
0.42 to 0.48	3303	3.65%
0.48 to 0.54	2489	2.75%
0.54 to 0.60	1911	2.11%
0.60 to 0.66	1427	1.58%
0.66 to 0.72	1196	1.32%
0.72 to 0.78	898	0.99%
0.78 to 0.84	760	0.84%
0.84 to 0.90	576	0.64%
0.90 to 0.96	456	0.50%
0.96 to 1.02	330	0.36%
1.02 to 1.08	265	0.29%
1.08 to 1.14	208	0.23%
1.14 to 1.20	154	0.17%
1.20 to 1.26	114	0.13%
1.26 to 1.32	108	0.12%
1.32 to 1.44	110	0.12%
1.44 to 1.62	80	0.09%
1.62 to 2.30	108	0.12%
2.30 to 3.80	100	0.11%
3.80 to 6.00	60	0.07%

D = spatial displacement changes between two consecutive time points. N = The frequency of occurrences within the transportation time interval.

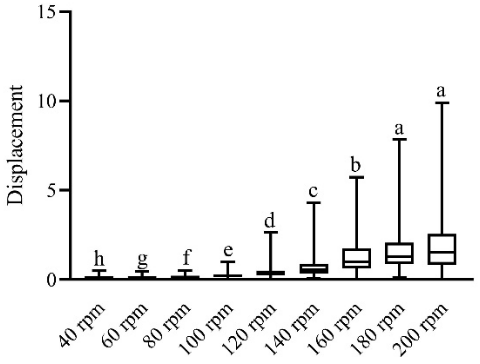

Figure S1. The displacement levels corresponding to different rotational speeds. By employing vibration sensors to measure the occurrence of vibrations in the laboratory orbital shaker, we utilize the spatial displacement changes between two consecutive time points to represent the intensity of the vibrations. Results are represented as box-plots with the highest and lowest values represented by whiskers, inter-quartiles between quartiles 1 and 3 shown as boxes, and median values. a–h: boxes with different letters differ significantly ($p < 0.05$).

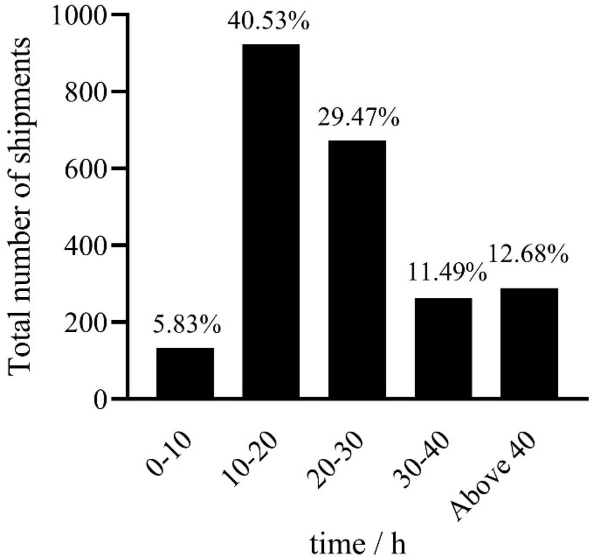

Figure S2. Semen delivery data (totally 2238 delivery data) from five boar stations in various regions of China between the year 2022 and 2023.

Table S2. Effect of the vibration emissions on sperm motility parameters. With a rotational speed of 200 rpm, sperm were processed for different durations (0, 3 and 6 h). Subsequently, during the storage of the semen (24, 48,72, and 96 h), the influence of vibrations on sperm motility was assessed through CASA; n = 15 boar

Parameter	Time (h)	0 h	24 h	48 h	72 h	96 h
	0	93.5±5.6^{a}	91.4±5^{a}	85.7±3.1^{ab}	81.6±3.5^{bc}	75.2±2.9^{c}
VCL (μm/s)	3	94.3±2.2^{a}	59.2±5.2^{b}	57.9±1.6^{b}	58.6±4.8^{b}	58.5±4^{b}
	6	92±6.8^{a}	55.6±1^{b}	52.2±0.4^{b}	55.7±4.1^{b}	55.9±3.3^{b}
	0	28.2±2.8^{a}	26.7±1.4^{ab}	25.8±0.2^{ab}	24.1±1.6^{b}	26.8±1.3^{ab}
VSL (μm/s)	3	27.9±3^{a}	20.4±0.6^{b}	17.2±1.2^{b}	19.2±1.1^{b}	19.7±0.7^{b}
	6	27±0.9^{a}	15.8±0.9^{b}	19.9±0.5^{b}	18.4±4.5^{b}	17.2±0.9^{b}
	0	49.9±6.3^{a}	47.8±2.3^{ab}	44.9±2.8^{ab}	40.8±1.2^{b}	43.7±4.1^{ab}
VAP (μm/s)	3	46.9±9.2^{a}	36.7±3.4^{b}	27.5±1.2^{b}	33.6±2.1^{b}	32.6±1.3^{b}
	6	48.4±6.4^{a}	25.4±0.6^{b}	28.6±0.6^{b}	29±4.6^{b}	27.5±0.6^{b}
LIN (%)	0	30.1±1.1^{a}	29.2±0.6^{b}	30.2±1.2^{b}	29.5±0.9^{b}	35.7±0.6^{b}

	3	31.7±3.9	34.7±2.2	29.6±1.5	32.8±2.3	33.8±2.3
	6	31.2±1.9[a]	28.4±2.2[ab]	28.1±0.8[ab]	28.1±2.2[ab]	25.8±3.7[b]
	0	56.7±4.1	56.1±5.2	57.7±4.1	59.1±3	61.5±3.3
STR (%)	3	57.4±3.4[ab]	56.1±4[b]	62.4±1.9[a]	57.2±0.4[ab]	60.7±2.3[ab]
	6	54.6±5.5[b]	61.9±2.3[ab]	69.6±1.1[a]	61.6±7.6[ab]	62.4±4.5[ab]
	0	53.3±4.8[ab]	52.4±5.1[ab]	52.4±3[ab]	50.0±1[b]	58.2±4.3[a]
WOB (%)	3	59.7±5.2[ab]	61.9±1.7[a]	50.8±5.5[b]	57.4±3.9[ab]	55.8±6.1[ab]
	6	54.7±1.8	45.8±1.8	54.8±0.8	51.9±6.2	49.3±1.8
	0	5.9±0.4[a]	5.7±0.9[a]	5.2±0.2[ab]	5.4±0.6[ab]	4.7±0.3[b]
ALH (μm)	3	6.0±0.7[a]	3.7±0.3[b]	4.3±0.1[b]	3.9±0.2[b]	4.3±1.1[b]
	6	6.1±0.6[a]	4.4±0.2[ab]	3.6±0.1[c]	4.7±0.3[b]	4.2±0.6[ab]
BCF (Hz)	0	4.2±0.2	4.5±0.6	5.1±0.2	4.7±0.8	4.8±1.1
	3	4.1±0.4[b]	5.1±0.4[ab]	5.6±0.7[a]	5.0±0.2[ab]	5.0±1.4[ab]

	6	4.1±0.8^{c}	5.5±0.2^{ab}	6.4±0.4^{a}	4.8±0.6^{bc}	5.3±0.2^{b}

Values are expressed as mean ± standard deviation. a-c Different letters within the same column are significantly different ($p<0.05$). A-C Different letters within the same low are significantly different ($p < 0.05$).

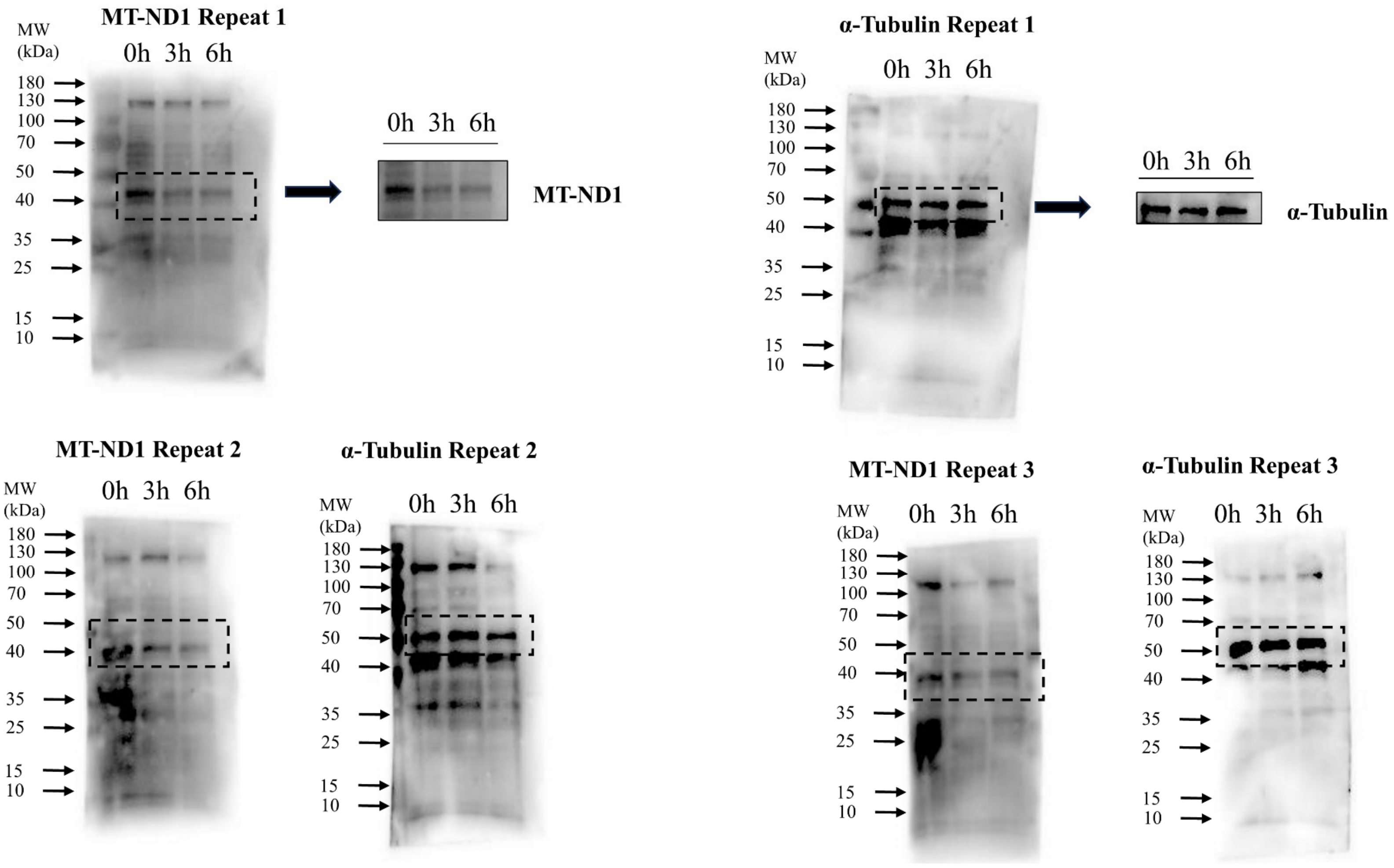

Figure S3. The original western blotting images showing the levels of mitochondria-encoded proteins MT-ND1 and α-Tubulin.

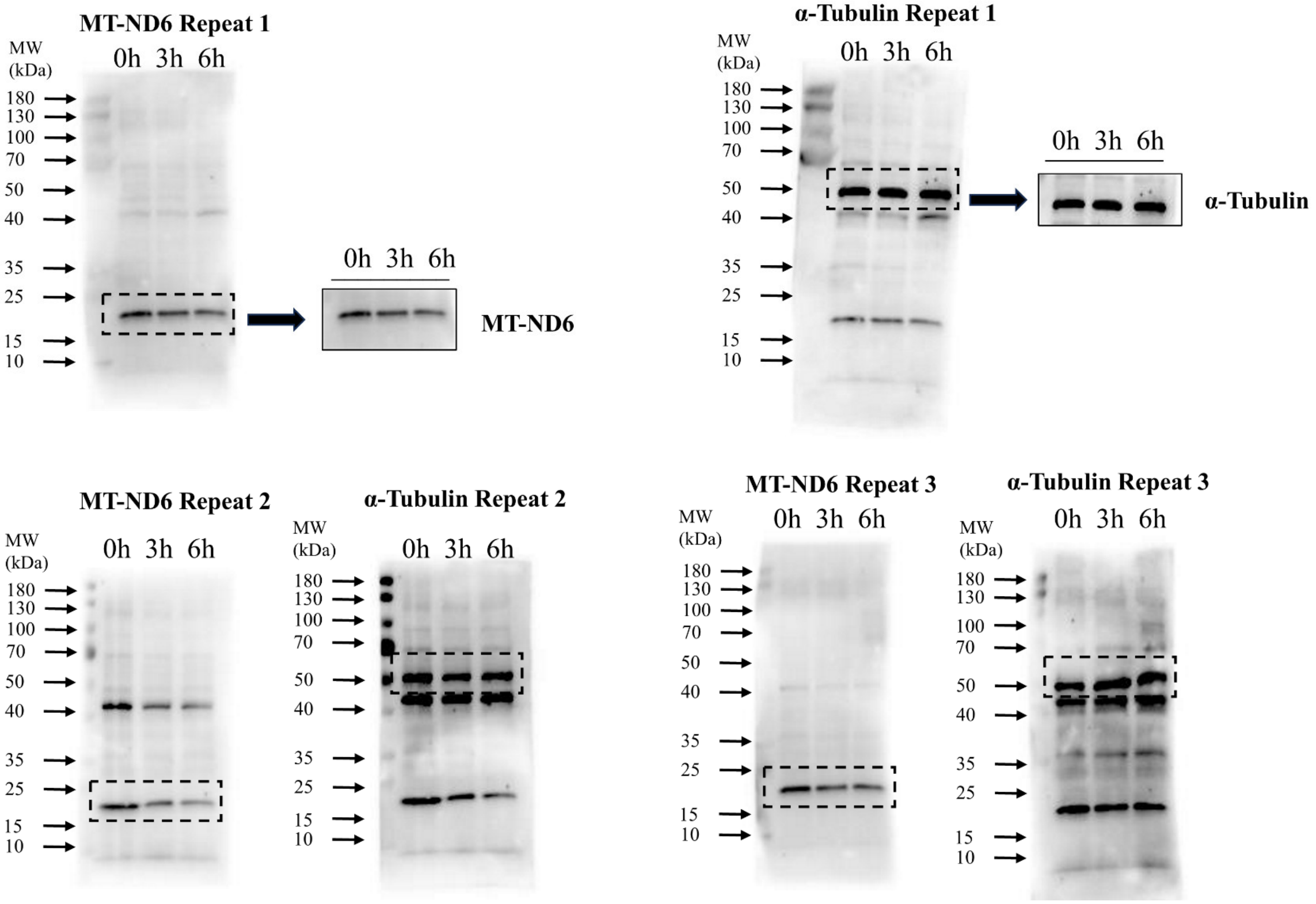

Figure S4. The original western blotting images showing the levels of mitochondria-encoded proteins MT-ND6 and α-Tubulin.

Tyrosine phosphorylation Repeat 1

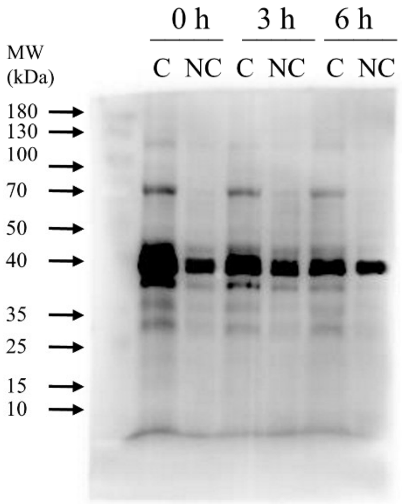

α-Tubulin Repeat 1

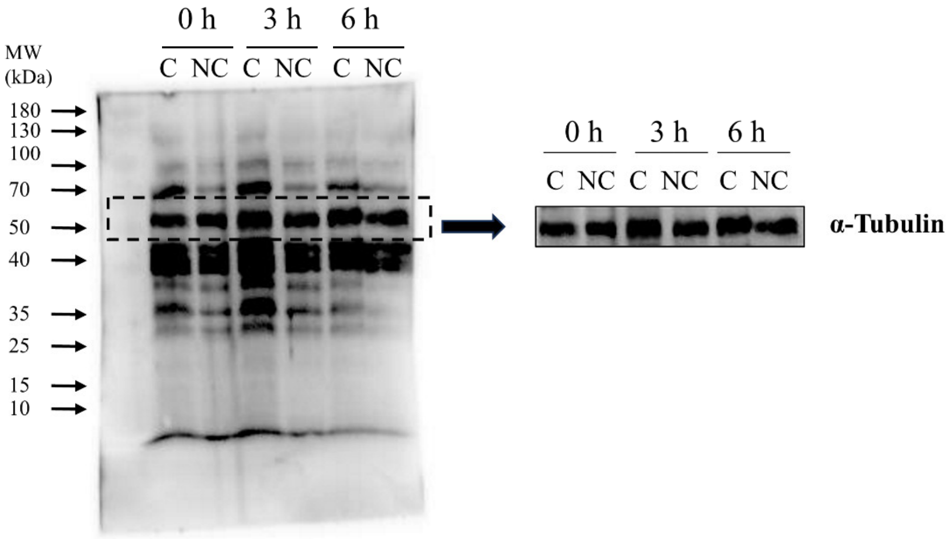

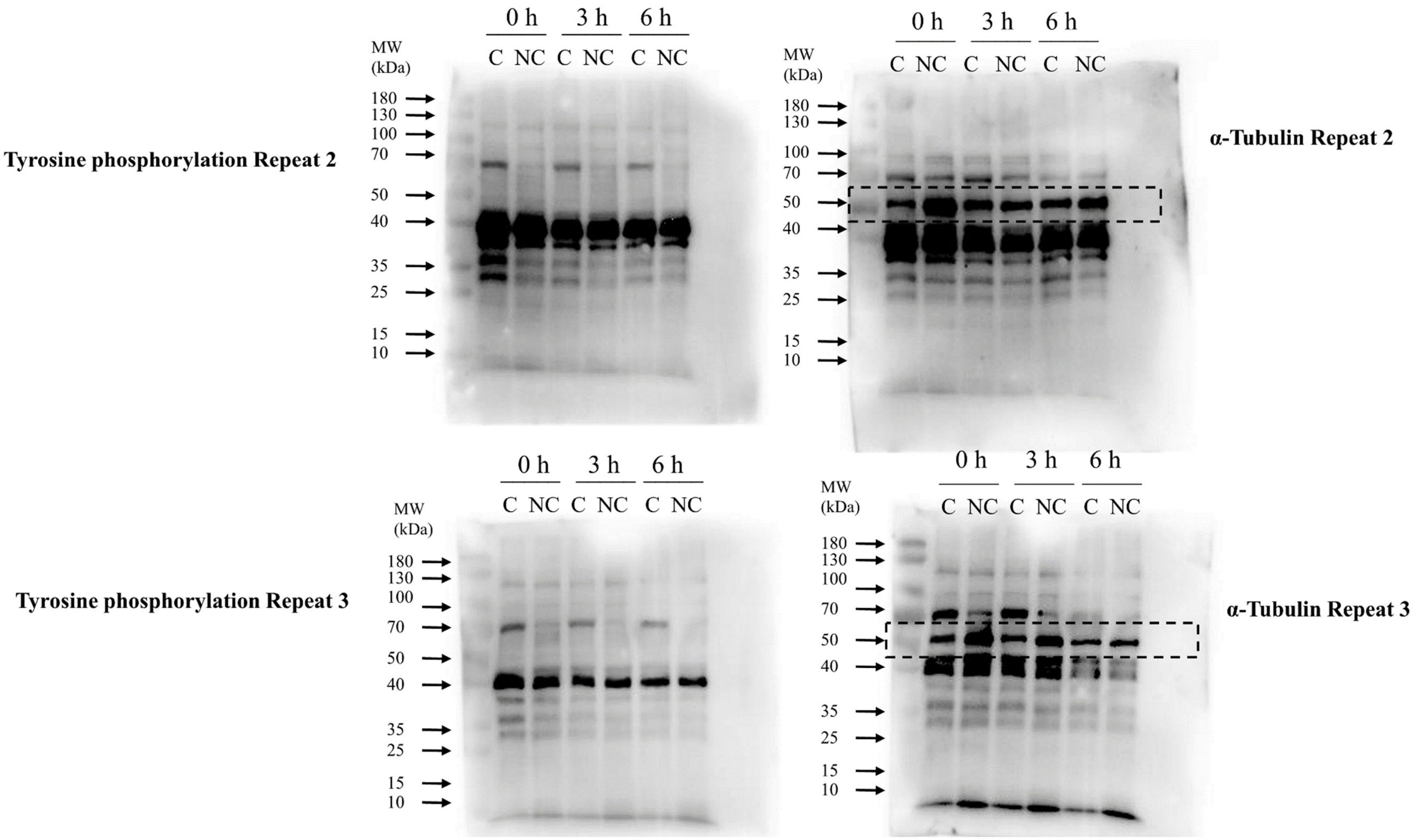

Figure S5. Effects of vibration emissions on sperm tyrosine phosphorylation level and α-Tubulin.

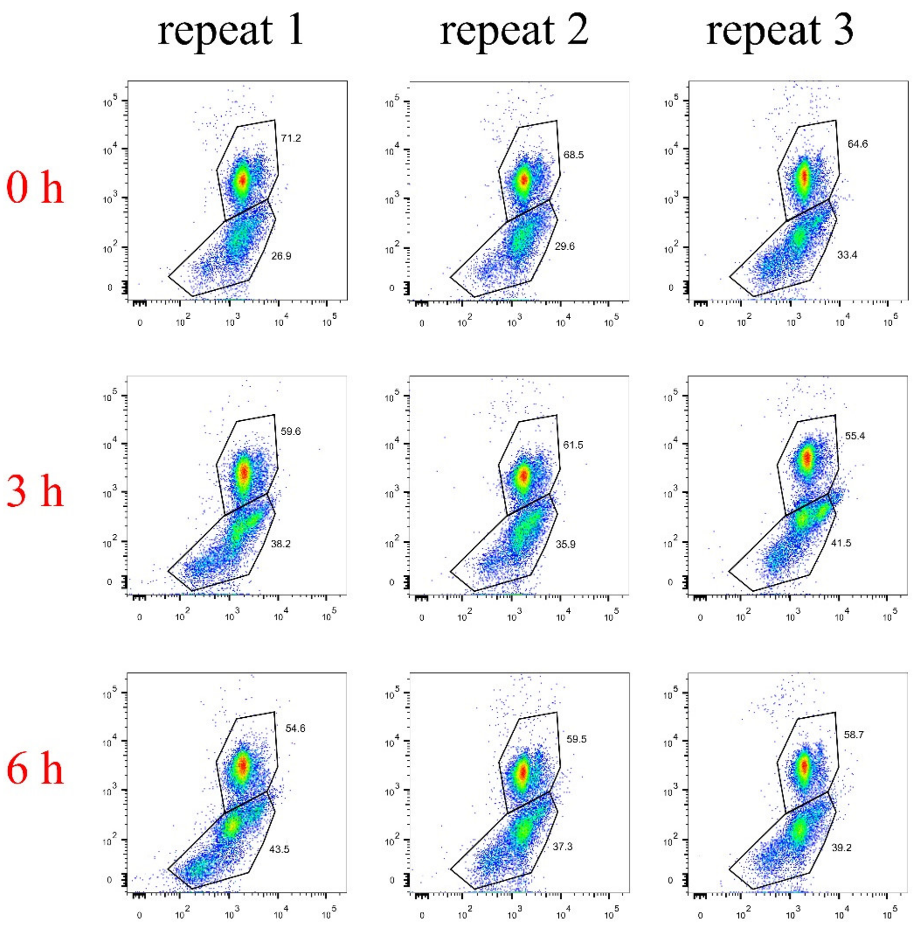

Figure S6. Effects of vibration emissions on sperm mitochondrial membrane potential.
